# The Influence of Region of Interest Heterogeneity on Classification Accuracy in Wetland Systems

**DOI:** 10.3390/rs11050551

**Published:** 2019-03-06

**Authors:** Tedros M. Berhane, Hugo Costa, Charles R. Lane, Oleg A. Anenkhonov, Victor V. Chepinoga, Bradley C. Autrey

**Affiliations:** 1 Pegasus Technical Services, Inc., c/o U.S. Environmental Protection Agency, Cincinnati, OH 45219, USA; 2 Direção-Geral do Território, 1099-052 Lisbon, Portugal; 3 Office of Research and Development, U.S. Environmental Protection Agency, Cincinnati, OH 45268, USA; 4 Laboratory of Floristics and Geobotany, Institute of General and Experimental Biology SB RAS, Ulan-Ude 670047, Russia; 5 Laboratory of Physical Geography and Biogeography, V.B. Sochava Institute of Geography SB RAS, Irkutsk 664033, Russia; 6 Department of Botany, Irkutsk State University, Irkutsk 664003, Russia

**Keywords:** general linear model (GLM), gerrymandering, Lake Baikal, methods, multinomial linear model (MLM), mixed pixels, random forest (RF), Selenga river delta, support vector machine (SVM), Worldview-2

## Abstract

Classifying and mapping natural systems such as wetlands using remote sensing frequently relies on data derived from regions of interest (ROIs), often acquired during field campaigns. ROIs tend to be heterogeneous in complex systems with a variety of land cover classes. However, traditional supervised image classification is predicated on pure single-class observations to train a classifier. This ultimately encourages end-users to create single-class ROIs, nudging ROIs away from field-based points or gerrymandering the ROI, which may produce ROIs unrepresentative of the landscape and potentially insert error into the classification. In this study, we explored WorldView-2 images and 228 field-based data points to define ROIs of varying heterogeneity levels in terms of class membership to classify and map 22 discrete classes in a large and complex wetland system. The goal was to include rather than avoid ROI heterogeneity and assess its impact on classification accuracy. Parametric and nonparametric classifiers were tested with ROI heterogeneity that varied from 7% to 100%. Heterogeneity was governed by ROI area, which we increased from the field-sampling frame of ~100 m^2^ nearly 19-fold to ~2124 m^2^. In general, overall accuracy (OA) tended downwards with increasing heterogeneity but stayed relatively high until extreme heterogeneity levels were reached. Moreover, the differences in OA were not statistically significant across several small-to-large heterogeneity levels. Per-class user’s and producer’s accuracies behaved similarly. Our findings suggest that ROI heterogeneity did not harm classification accuracy unless heterogeneity became extreme, and thus there are substantial practical advantages to accommodating heterogeneous ROIs in image classification. Rather than attempting to avoid ROI heterogeneity by gerrymandering, classification in wetland environments, as well as analyses of other complex environments, should embrace ROI heterogeneity.

## 1. Introduction

Remote sensing plays a significant role in environmental monitoring and management applications, helping to quantify the effect of both natural and anthropogenic factors on the spatiotemporal dynamics of wetland systems [1]. Fundamental remote sensing data-processing workflows include the acquisition of applicable remote sensing data, implementation of a systematic and appropriate field data collection procedure, and selection and calculation of data-analysis algorithms that meet the current state of knowledge and the scope of the research being undertaken. The effective integration of this process creates highly accurate, repeatable, and relevant geospatial products [2].

Myriad possible combinations of spatial scales and analytical methods make effective execution of any remote sensing project a daunting task [3-6]. For example, the increasing spatial and spectral resolution of economically affordable remote sensing data challenges fundamentals of remote sensing, such as delineation and sampling of representative plots that are spectrally homogeneous for field data collection [7,8]. Yet higher heterogeneity of spectral responses is often expected in natural environments, particularly with the advance in sensor capabilities [9]. This leads to a paradox: increased spatial and spectral resolution promising new opportunities for remote landscape classification can discern an abundance of unique end-product classes requiring highly demanding and costly field efforts (i.e., an abundance of field sampling points) and the derivation of spectrally homogeneous regions of interest (ROIs) such that many of the advantages of using remotely sensed data could be lost.

Feature extraction and classification of natural objects with heterogeneous spectral signatures (e.g., mixed ROIs, composed of pixels with differing class membership) often result in erroneous classification in the final thematic map if not dealt with conveniently. For example, probabilistic functions underpinning traditional supervised classifiers assume ROI purity [10,11], and large samples of pure ROIs are suggested for better classification accuracy outcomes in training supervised classifiers [7,12,13]. Consequently, end-users frequently opt to create pure or homogeneous ROIs (e.g., moving ROIs away from field-based points, or gerrymandering the ROI), potentially inserting error into their classification. The feasibility of creating pure ROIs in the field, especially in light of the aforementioned satellite resolution and spectral bandwidth paradox, is likely to be limited by the required amount of financial, logistical, and time resources [13,14]. This is particularly true when trying to collect large and spectrally homogeneous representative data in large wetland systems due to the high diversity of wetland classes and plant species composition per unit surveying area [15], driven by manifest differences in hydropatterning (at coarser spatial scales) and hydroperiod (at finer spatial scales). For example, the hydrogeomorphology of deltaic wetland ecosystems can be unique and complex with rivers meandering through the delta creating both depressions (e.g., pools) and elevated terraces (e.g., river banks and islands), with great heterogeneity of edaphic conditions reflected in high local and landscape-scale diversity. Nevertheless, researchers have pushed to establish baseline data on wetland systems across the globe, such as Russia’s Selenga River Delta into Lake Baikal [16,17] or Botswana’s Okavango Delta [18] through the judicious use of emerging classification approaches (e.g., [19]) and technological advances (e.g., [20,21], see also [2,22] for the current state-of-the-science in wetland satellite remote sensing).

It is against this backdrop that there is increasing utility and emerging need to conduct classifications using heterogeneous training ROIs. Classification of ROIs with mixed spectral response involves computation of the wetland class end member proportions by ROIs rather than through probabilistic determination of the spectral response of a pixel to a particular wetland class end member. Many studies have shown the potential advantages of using mixed ROIs in training (e.g., [4,13]). Brown et al. [12] stated class mixture modeling is fundamental and suggested a discrimination approach when discrete class densities do not overlap in feature vector space and statistical pattern recognition methods when they do overlap. Amancio et al. [23] noted that despite the long tradition of using pattern recognition algorithms in applications such as industry, commerce, and academic research, there is no single method that yields the best accuracy results. Despite these admonitions, classification with mixed-ROIs remains uncommon.

With the increasing number of high-resolution satellite sensors creating a paradox for effectively characterizing ecosystems, we sought to answer the following research question: Do mixed ROIs yield better classification accuracy in complex remote sensing classification approaches? In addressing this research question, we used four classifiers often trained with samples of only single-class observations, but also allowing for heterogeneous multi-class observations. Specifically, we used and contrasted both parametric and nonparametric approaches: (1) Multinomial Logistic Model (MLM), (2) Generalized Linear Model (GLM), (3) Support Vector Machine (SVM), and (4) Random Forest (RF). The literature we assessed (e.g., [4]) and the paradox we observed suggested that mixed ROI approaches would outperform pure ROI approaches. We conducted our research in a large freshwater deltaic wetland with high complexity (i.e., with ~22 discrete classes of wetland and open-water habitats [17,24]). Our goal in conducting these unique and novel analyses was to assess and characterize the benefits and detriments of using mixed-ROIs to provide useful information for end-users in selecting and parametrizing wetland classification models.

## 2. Materials and Methods

### 2.1. Study Area

**Figure 1.**
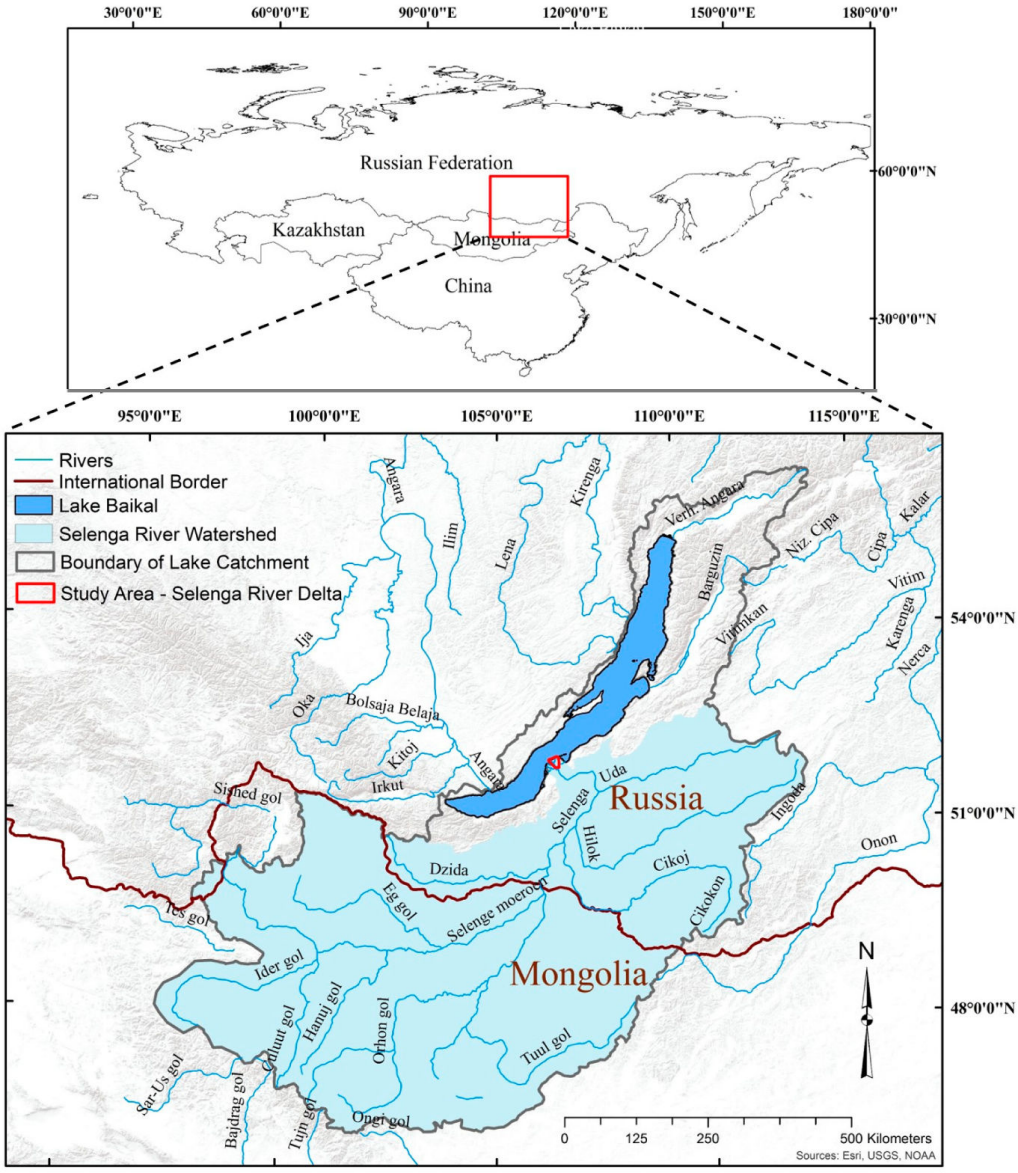
The location of the study area of the Selenga River Delta, a large, freshwater deltaic wetland extending into Russia’s Lake Baikal.

The study area is the Russian Federation’s Selenga River Delta located in southern Siberia (Figure 1). The hydro-climate of the region is characterized by high-amplitude daily and seasonal air temperature fluctuations, cold and long winters, short springs and short but warm and relatively rainy summers [25,26]. The delta covers an area of ~1100 km^2^ at the terminus of the Selenga River, the major fluvial contributor of water, sediment and contaminant inflows to Lake Baikal [27]. The Selenga River contributes 50–60% of the total inflow to Lake Baikal [28-31], the oldest (20–25 million years) and deepest (>1600 m) lake in the world. Lake Baikal contains 26% of the water volume of the world’s freshwater lakes, and ~6% of all global fresh water resources, including lakes, rivers, glaciers, etc. [32]. Recognized as a World Heritage Site by United Nations Cultural and Educational Organization (“UNESCO”), the Selenga River Delta into Lake Baikal is home to a wide variety of flora and fauna [31]. The delta acts as a buffer for attenuating and removing anthropogenic contaminants originating from the Selenga River Basin, an area of ~450,000 km^2^, before reaching Lake Baikal. The density of wetlands and small channels of the delta have shown steady and significant contaminant (e.g., metals and persistent toxic organic compounds) removal under various flow regime conditions [28,33,34]. With an increasing pressure on the eco-hydrology of the Selenga River in general and the flora and the fauna of the river delta in particular, studying the delta’s wetland systems and aquatic habitats is paramount in an effort to preserve its vital ecosystem services and functions for the future [35,36].

### 2.2. Remote Sensing Data Acquisitions and Pre-Processing

Two overlapping cloud-free WorldView-2 images (WV2; DigitalGlobe, Westminster, CO, USA) were acquired in 2011 (images taken on 25 June and 3 July). WV2 has eight multispectral bands and one panchromatic band with 2.0-m and 0.5-m spatial resolutions, respectively. The eight multispectral WV2 bands include four newer bands (i.e., coastal, yellow, red-edge, and near infrared-2) in addition to the four “traditional” bands (i.e., blue, green, red, near infrared-1). These additional bands have been found to improve wetland vegetation and habitat discrimination and classification (e.g., [24]). The spatial extent of the study area covered by the two images is 215 km^2^, focusing on a central portion of the Selenga River Delta. Absolute radiometric calibration factor and effective band-width values provided with the imagery metadata were used to evaluate the radiometric compatibility of the two images before the digital number (DN) values were converted to the top-of-the-atmosphere reflectance values in ENVI (v. 5.3, Exelis Visual Information Solutions, Inc., Harris Corporation, Broomfield CO, USA). Ortho-rectification was not necessary since the two images are Ortho-Ready Standard (OR2A) with geo-accuracy error of <5 m based on 21 ground control points (GCPs) that were collected during the field data collection season (described below). The two images were mosaicked in ENVI and then classified into an initial 22 unsupervised classes as described in detail by Lane et al. [24] using Iterative Self-Organizing Data Analysis (ISODATA) clustering technique in ENVI. The unsupervised ISODATA wetland class thematic map produced was used for initial field data collection. The vector layer polygons created from the ISODATA classified image were loaded into a Trimble Nomad and/or a Trimble Yuma GPS receiver (Sunnyvale, CA, USA) with 2- to 5-m real-time accuracy for field data collection.

### 2.3. Field Data Collection and Processing

**Figure 2.**
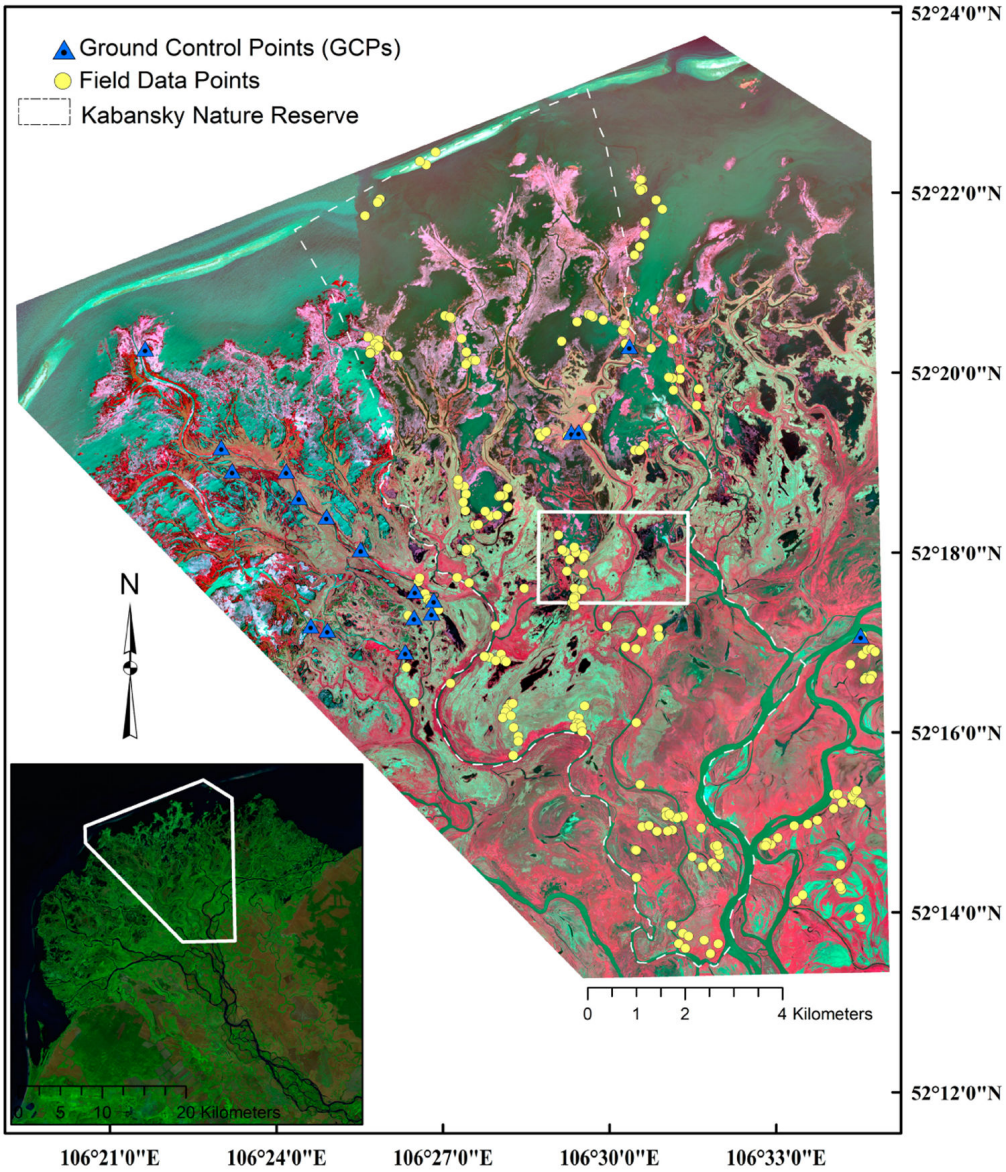
False color composite (NIR-1, Red and Green bands) of the focal study area within the Selenga River Delta and the location of the both the field collection points and ground truthing sites. The white-colored box indicates the location of an area of interest analyzed to generate wetland class and aquatic habitats classification thematic maps using the four classifiers.

**Figure 3.**
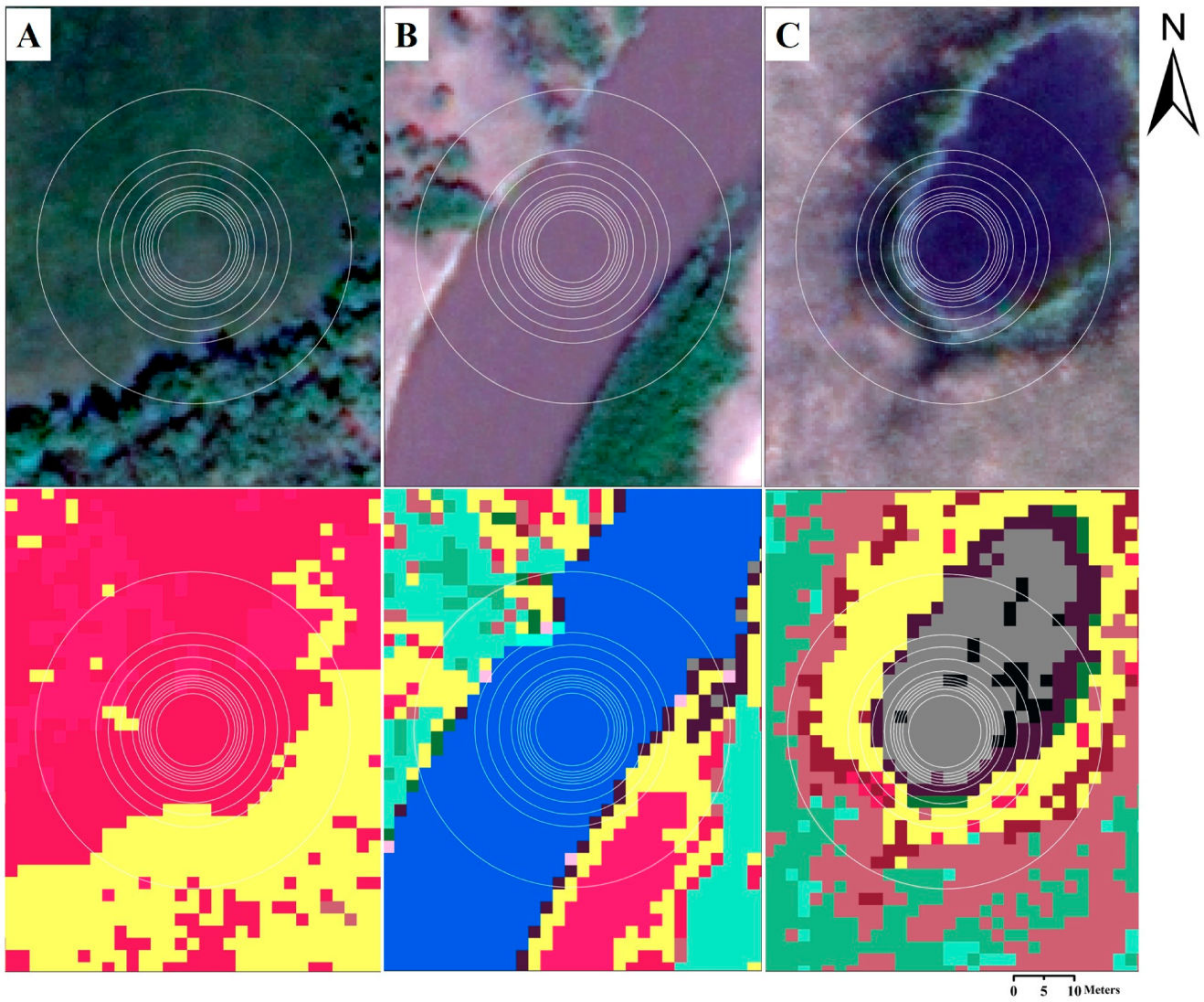
(**A–C**) Spatial delineation of ROIs with sample wetland classes and aquatic habitats selected from the Selenga River Delta landscape (Top panels are true-color images for **A**: predominantly terrestrial habitats; **B**: predominantly lotic habitats, and **C**: predominantly lentic habitats; see bottom-panel class descriptions in Table 1). The ROI diameters range from 12 to 52 m (see Table 2).

A total of 228 field sites and 21 GCPs in the focal area of Figure 2 were visited by boat, vehicle and foot in 2011 and 2012. A 100-m^2^ area typical of the target ISODATA class was chosen by the field team, and vegetation (i.e., species abundance occurring >10%) and corresponding habitat data were collected (see, e.g., [17]). Between three and 17 unique polygons were visited for each of the 22 classes (average: 10 field sites per class). These botanical data were subsequently collapsed to the genus phylogenic level and used to both train and validate the classifiers. Information on the vegetation composition and structure of the classes is available in Table 1. Circular ROI polygons of increasing diameters (Figure 3A-C) were delineated around each field site in ArcGIS (v. 10.4.1, ESRI Inc., Redlands, CA, USA). Differing diameters were arbitrarily chosen (from 12 m to 52 m; area ranged from 113 to 2124 m^2^), as there is no set distance for assessment, and end-users may choose to use any given diameter or shape, as dictated by the application at hand, such as field data collection designs. As expected, increasing the diameter increased the potential range of controlling factors affecting vegetation structure and type (e.g., increased the likelihood of different hydrologic regimes affecting vegetation composition). Ergo, increasing ROI heterogeneity resulted from increased ROI diameter length (Table 2). The relative purity of the ROI was also a function of the field site location. Field data collected from the center of large uniform features (e.g., a pond or a large river course) would be expected to maintain ROI purity with increasing ROI diameter (Figure 3A-C; see also Table 2).

### 2.4. Mixed-ROI Image Classification

Four classification algorithms (i.e., MLM, GLM, SVM, and RF) were implemented in R (R Core Team 2016) using the following packages: nnet [37], glmnet [38], gmum.r [39], and ranger [40]. Each classification algorithm was iteratively analyzed, using ROIs with 11 increasing diameters and hence increased heterogeneity (see Table 2). Heterogeneity of the ROIs was used to weight their contribution to the classification learning. That is, ROIs were used to provide information on the classes (among the 22 classes) whose membership was larger than zero (i.e., covering >0% of the ROI’s area). These membership degrees (i.e., cover percentages) were provided as observation weights to the classifiers, following [4]. For example, in R’s function ‘ranger’, the membership degrees were passed to RF via argument ‘case.weights’. To focus on the influence of ROI heterogeneity, the classification parameters other than heterogeneity were not modified between ROI sizes. WV2 has eight spectral bands and the WV2 spectral band mean and standard deviation from all pixels within each ROI were calculated, resulting in a total of 16 predictor variables.

#### 2.4.1. Multinomial Logistic Model

MLM is an extension of the binary logistic model to handle cases in which the variable of interest such as land cover can take multiple classes. In both MLM and binary logistic model approaches, the goal of the models is to describe the assumed linear relationship between the response variable and the predictors. MLM has been used in statistical analysis for decades and can be seen here as a conventional classifier normally trained only with known pure responses. However, the model can also accommodate mixed responses, for example through the use of an Artificial Neural Network (ANN). ANNs are an intelligent machine-learning algorithm approach initially developed to study biological functions and hence mimic the flight of energy along neural networks in the human brain, predicting outputs by processing non-linear and complex interactions using input-predictor variables [41]. A simple network without hidden layering can fit multinomial logistic models [37]. This specific net architecture has the advantage of accommodating mixed training units (i.e., mixed ROIs) in training, which is typically not possible in traditional statistical packages.

#### 2.4.2. Generalized Linear Model

GLMs are extensions of linear models by using a link function that relates the expected value of the response to a linear combination of the predictors. GLMs are suitable for modeling response variables of arbitrary distributions, including binary and count data. Here, GLMs were used to fit multinomial models similar to Section 2.4.1 above, but also with regularization and feature selection [38]. This strategy promotes robustness of modeling in classification, especially when the dimension of the data is large relative to the sample size, which is the case of the paradoxical scenario of increased spatial and spectral resolutions used in remote sensing applications.

#### 2.4.3. Support Vector Machine

SVM supervised nonparametric techniques produce high classification accuracy results with limited ground-truthed data by determining an optimum hyperplane separating the training dataset into discrete user-defined class end-members [13,42-45]. The SVM algorithms are particularly well informed by mixed ROIs and can be particularly robust when the hyperplane is located close to the center of class end-members ascribed by pure- and mixed-ROI training samples divided in feature space [13].

#### 2.4.4. Random Forest

RF is based on ensemble machine-learning and is increasingly being used as a classifier of choice for remote sensing analyses of different habitats (e.g., [16,46-48]). RF is nonparametric, and can be used for both classifications and regressions, as well as for determining variable importance [49,50]. In RF, a user-defined number of trees (ntree), each split at a node using input-predictor variables (mtry), contributes a single vote, where class end-membership assignment of the input vector is based on the majority of the votes. RF are constructed using a bootstrap aggregation approach where the input data are randomly selected with replacement to respectively train the trees (in-bag samples) and to perform internal accuracy assessment (out-of-the bag samples) [49,51].

### 2.5. Accuracy Assessment

Overall accuracy (OA), producer’s accuracy (PA), and user’s accuracy (UA) were assessed via Monte Carlo cross-validation (mean of 100 iterations), in which 75% of the total number of ROIs available (*n* = 171) were used for training the models while the remaining 25% (*n* = 57) were used for performing an independent classification accuracy assessment. The three accuracy measures are reported as the mean of the 100 iterations. We quantitatively assessed if the observed differences among the overall accuracies were statistically significant using 95% confidence intervals [52].

## 3. Results

**Figure 4.**
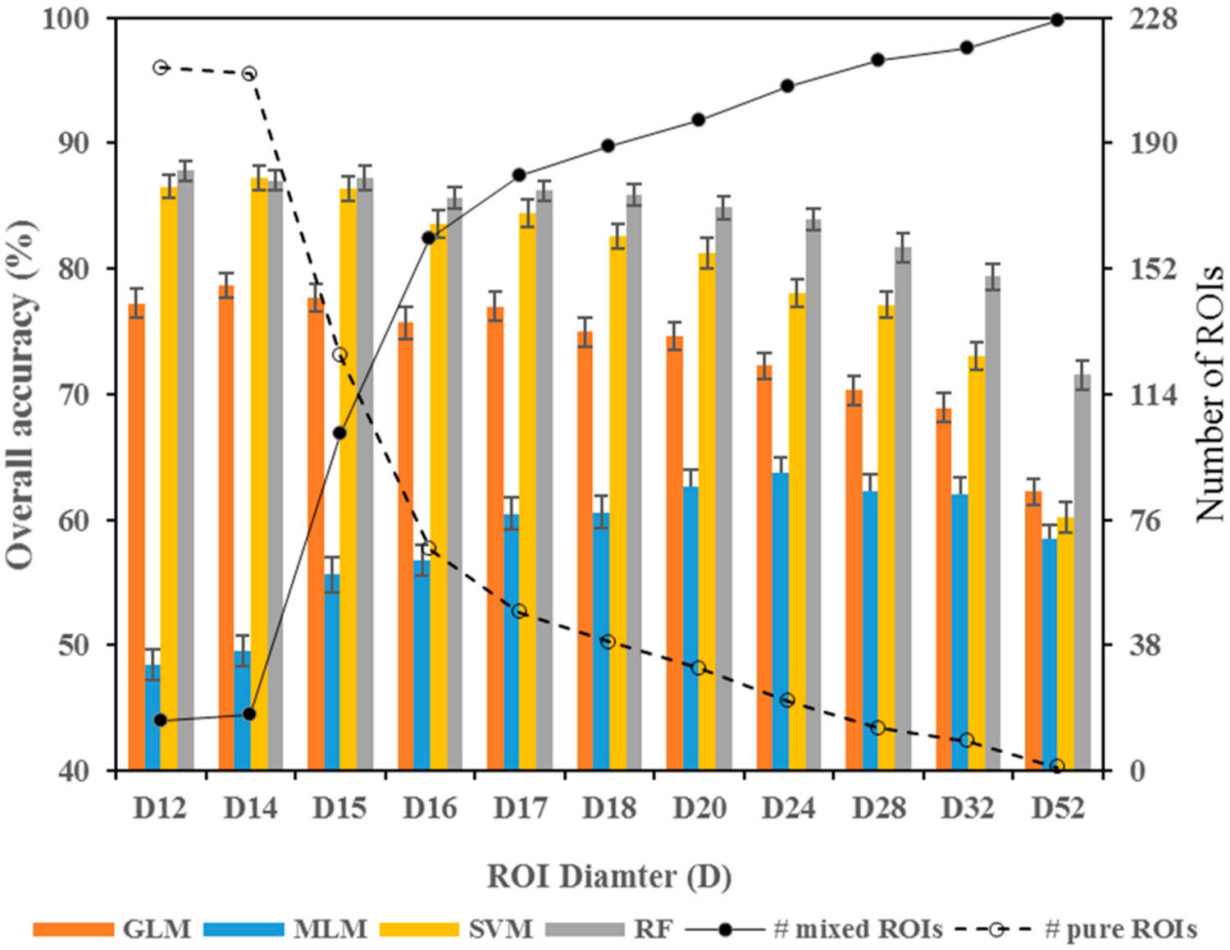
Overall accuracy and 95% confidence interval results by classifier type, by ROI size and the associated proportions of pure and mixed ROIs for the 228 sites in this study. Note: ROI = region of interest, GLM = Generalized Linear Model, MLM = Multinomial Logistic Model, SVM = Support Vector Machine, and RF = Random Forest.

**Figure 5.**
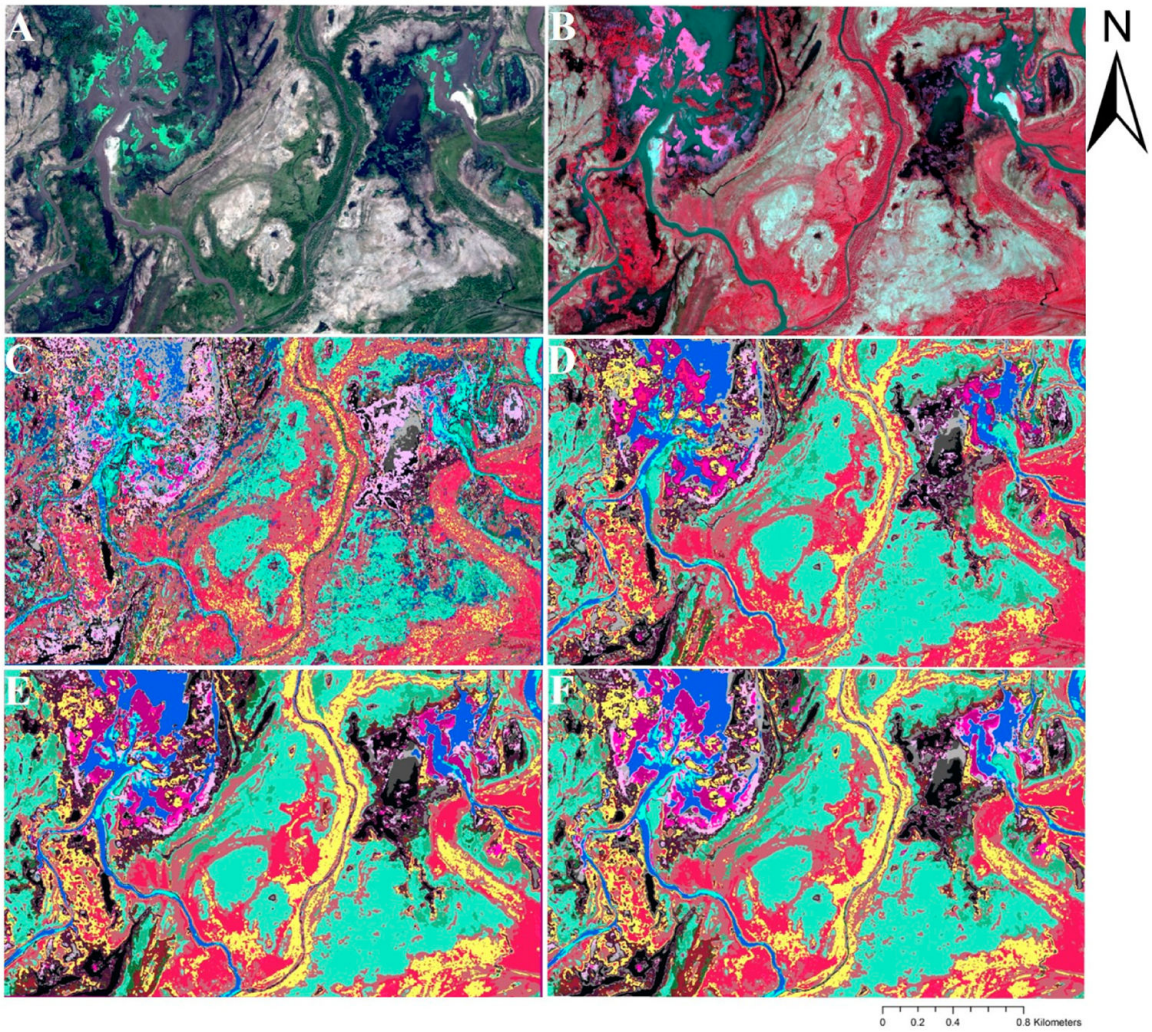
Wetland classification thematic maps of a select study area generated using the smallest ROI-size training data (ROI-ID D12; see Figure 2): (**A**) true-color WV2 bands 532, and (**B**) false-color WV2 bands 753 composite, (**C**) MLM, (**D**) GLM, (**E**) SVM, and (**F**) RF models. Colors depicted follow the wetland classes given in Table 1.

The highest and lowest OA (87.8% and 48.4%) were achieved using RF and MLM, respectively, in both cases with the smallest ROI-size (Table 3; Figure 4). RF outperformed the other classifiers (except for ROI-ID D14), followed closely by SVM, before the performance of SVM decreased well below that of RF for extreme heterogeneity. The GLM performed moderately well, with OA ranging from 62–79%, with better performance at smaller ROIs. Interestingly, unlike the other classifiers, MLM performed poorly at smaller ROIs and increased in performance through ROI-ID D24 (64%) until OA began decreasing again. An example of the differences in classification application between the algorithms may be seen in Figure 5, in which the map of the MLM presents a highly pixelated structure (Figure 5C) compared to the remaining maps, relatively more consistent among them (Figure 5D-F).

The OA changes vacillated with ROI size and, implicitly, by quantified heterogeneity (see Table 2) in the range of |0.9%–1.5%| for the three best-performing classifiers (with the exception of the largest ROI size). Significant differences arose once ROI-ID D15 (area = 177 m^2^) was exceeded (Table 3). Further increasing ROI heterogeneity to ROI-ID D17 (227 m^2^) afforded an improvement in RF and GLM OA that again made the differences between those approaches statistically insignificant. Exceeding D17 (227 m^2^) made the differences statistically significant for the three classifiers. However, OA decreased smoothly until ROI heterogeneity was at a maximum. As for MLM, its performance lagged as measured by the OA yet MLM had the least change in OA across the ROIs (averaging |1.0%|). The stability of MLM is suggestive of potential utility, but that is belied at this point by the low OA, particularly marked at small ROI sizes.

**Figure 6.**
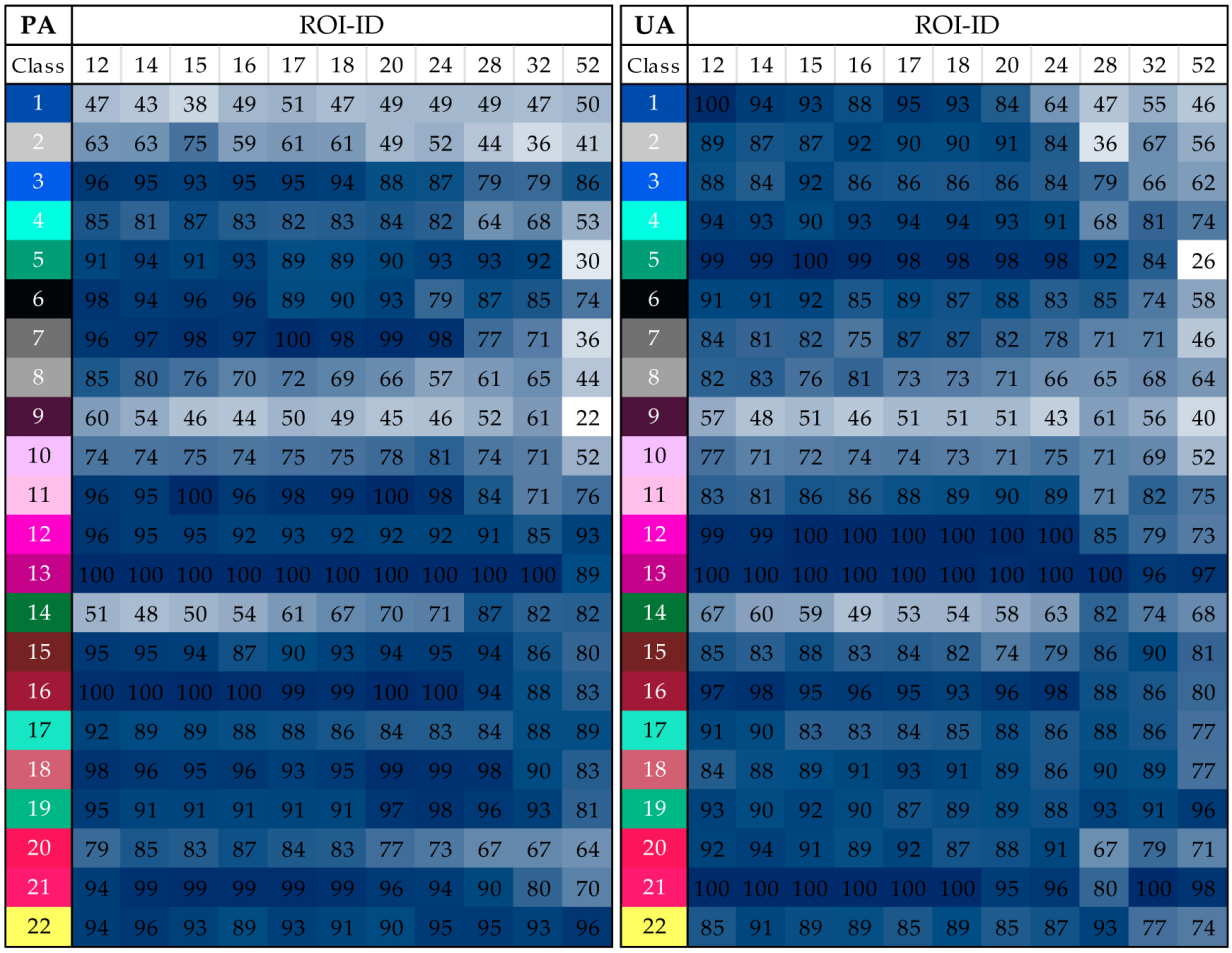
Producer’s accuracy (PA) and user’s accuracy (UA) of the 22 classes by Random Forest and ROI size. PA and UA values are colored in blue as a function of their magnitude (darker colors represent higher values). The class columns are colored as in Table 1.

Per-class accuracy estimates were also produced, and the producer’s and user’s accuracy for each class by the best-performing classifier (RF) are provided in Figure 6. The classes mapped with larger accuracy, both in terms of PA and UA, were classes 12 (Dense floating vascular (*Nymphoides*)), 13 (Very dense floating vascular), 16 (Persistent emergent (*Equisetum*)) and 21 (Persistent terrestrial (*Amoria*)). These and most of the classes followed a decreasing trend of accuracy as ROI size increased. The largest PA and UA values were obtained across several ROI heterogeneity levels (e.g., PA of class 13 reached 100% up to ROI-ID D32). The smallest PA and UA values (22% and 26%) were obtained with the largest ROI size (D52) for classes 9 (Submerged floating vascular (Nymphoides)) and 5 (Shallow water with sand bottom), respectively. Presenting an inverse trend was class 14, a monoculture-forming type of Persistent emergent (*Phragmites*). This class apparently benefited from ROI heterogeneity for enhanced per-class classification accuracy. Class 9 (*Nymphoides*) was somewhat insensitive to ROI heterogeneity, and hence its PA and UA were relatively flat across all ROI sizes (except ROI-ID D52).

## 4. Discussion

Lewis Carroll’s Red Queen notes, “Now, here you see, it takes all the running you can do, to keep in the same place. If you want to get somewhere else, you must run at least twice as fast as that!” [53]. Advances in remote sensing imagery analysis and increased availability of sensed electromagnetic bands requires us to “move faster” (i.e., more creatively analyze the data) to effectively classify landscapes. The recent development and application of machine-learning classifiers such as ANNs, SVM, and RF approaches have quickly (pun intended) advanced our efforts to understand landscape patterning using spectral data. The science has moved from early supervised classification efforts (e.g., using Maximum Likelihood (ML); [54,55]) to using RF, SVM, ANN, and other approaches. However, the utility of the advanced methods when confronted with non-homogeneous ROIs in wetland classification has remained problematic (resulting in gerrymandered ROIs or data point nudging), insofar as our literature analyses have discerned.

Some authors have included wetlands as a discrete class when contrasting among methods, though none have wetland systems as diverse (e.g., with 22 wetland classes) as in our analyses. For instance, classifying seven land cover types (citrus, pasture, sod, timber, urban, water and wetland) using Landsat-5 TM data, Dixon and Candade [56] reported OA of 51%, 78% and 79% using ML, ANNs and SVM, respectively, concluding SVM is faster and easier to implement than ANNs. Moreover, Foody and Mathur [13] found comparable OA using conventional pure-pixels (92.6%) and mixed-pixels (91.1%) classification approaches using unbalanced training datasets, 3-band SPOT HRV multispectral data and SVM to classify three agricultural cover types.

In our analyses, we increased ROI size up to 2124 m^2^. As the ROI size increased, the number of different wetland class end-members defined by the boundaries of the ROI also increased (e.g., Figure 3). As we note, mixed-ROIs will become increasingly commonplace with technological advances. Across our analyses, though, ROI sizes increased nearly 19-fold, yet accuracies with RF (and to a lesser degree, SVM) stayed remarkably consistent, even as the ROI heterogeneity increased. To wit, we achieved OA of nearly 80% with RF (ROI-ID D32, 804 m^2^, eight times our field-based quadrat size of 100 m^2^) even though 96% of the ROIs were heterogeneous. We developed our approach using 22 classes, and it is likely that fewer wetland classes would have resulted in even greater OA across the different classifiers (e.g., [19]).

Across all ROI sizes and with increasing heterogeneity, both SVM and RF were superior to MLM and GLM (though we note the choice of classifier can be contextually dependent). The poor performance of MLM for small ROI sizes is perhaps explained in part by the small representativeness of some classes in the training samples. Since 228 ROIs were used to train 22 classes, and most of the ROIs were pure, there were on average only 228/22 ROIs per class (~10). This is a small sample size for training multinomial logistic models given the number of predictors (16). Note that ANNs are able to perform well with small sample sizes, but in this case they were used to fit multinomial logistic models. Increasing the size of the ROI increases heterogeneity and thus each individual ROI provides information on a larger number of classes (as many classes as it encloses). Increasing ROI size and heterogeneity can be seen as an indirect way of increasing sample size. Possibly for this reason, the OA of MLM increased with increasing diameter up to 24 m. That is, the particular behavior of MLM increasing OA from 48.4% to 60.6% was possibly caused by the richer information held in the mixed ROIs, which increases the number of ROIs available for training individual classes.

GLM, also used to fit multinomial models, was less sensitive to the small representativeness of the classes when the ROIs were small and mostly pure, possibly due to regularization and feature selection [38]. Therefore, traditional classifiers such as those with a statistical basis, which commonly require large samples, can perform relatively well in sub-optimal conditions, as long as some refinement is implemented. However, nonparametric algorithms are by design not constrained to use linear combinations of the predictors, and this may be advantageous. Thus, SVM and RF achieved higher OA.

While both SVM and RF allow integration of ancillary data, we concur with others (e.g., [42,57-59]), and recommend RF due to the need for lower parametrization, informative generation of variable importance (i.e., Mean Decrease in Gini values), detection of outliers, and construction of simple decision boundaries. When contrasted with RF, SVM is computationally costlier and less accurate than RF, particularly when an abundance of predictor variables (i.e., hyperspectral or multi-source data) are utilized [49]. On the other hand, RF requires a veritable forest of trees (i.e., a large number) to train the model, and SVM demands abundant support vectors to build its model. Furthermore, perhaps based on the limited number of parameters to select with RF or our familiarity with the approach (e.g., [16]), we may have optimized the RF results relative to SVM.

Irrespective of the classifier used, OA was stable across several ROI sizes. For example, the 95% CI of the largest OA for RF, obtained with ROI-ID D12, overlaps that of larger sizes (see Table 3). This means that there is no statistical significance of the difference observed between the OA for a range of ROI diameters. Table 2 shows that the percentage of pure ROIs decreased from 93.4% to 21.1%. Despite this big difference in ROI heterogeneity as a function of their size, the differences in OA are statistically insignificant. Similar situations are observed for GLM and SVM. Per-class classification accuracy also suggest strong ability of the classifiers to hold varying degrees of ROI heterogeneity. The producer’s and user’s accuracies of most of the classes stayed relatively high across several ROI sizes. There was only one clear exception (class 14), which benefited from ROI heterogeneity. Possible reasons for this distinct behavior of a monoculture-forming vegetative class (*Phragmites*) are not clear, which merits further research. Nevertheless, none of the classes seemed to suffer from ROI heterogeneity until extreme levels were used. Our findings, then, suggest that there are substantial practical advantages to creating mixed-ROIs rather than gerrymandering. In classification, the only thing that needs to be done is to recognize the heterogeneous nature of the ROIs rather than assuming they are necessarily pure, and use their heterogeneity directly in training the classifiers. The suggested way of using heterogeneity in training is to find the relative proportion of the classes found in the individual ROIs and use it as class membership (training weights), which is included in the settings passed to the classifiers [4].

The OA of GLM, SVM, and RF decreased significantly (i.e., the confidence intervals ceased overlapping) within increasing ROI size and hence heterogeneity, as compared to their respective highest OA (at D14, D14, D12, respectively; see Table 3). Where the OAs ceased overlapping, for ROI-ID D16 (for SVM) and D18 (for GLM and RF) the percentage of pure ROIs decreased to 29.4% and 17.1%, respectively. Therefore, we found classification accuracy starts decreasing significantly when the percentage of pure ROIs crosses a certain threshold. This suggests that there is a limit of the heterogeneity the classifiers can accommodate in training before their performance degrades. Recent research has shown that both mixed and pure training responses are needed. Classifiers can learn from pure responses to describe the classes and from mixed responses to separate the classes [4,13]. Ma et al. [60] found that the ratio of pure to mixed training units should lie between 0.2 to 0.6 for their dataset, which matches the ratios defined by ROI-ID D15 to D17. However, the specific threshold should fluctuate as a function of the application, including study area, number of classes, classifier, etc.

## 5. Conclusions

The world’s wetlands are highly diverse in vegetative and habitat structure, and that diversity creates hot spots of biodiversity and functioning. Yet wetland losses around the world continue (e.g., [61]), and these will be exacerbated by expected changes in precipitation patterning and timing (e.g., [62]). Satellite remote sensing is a cost-effective approach to creating baseline understanding of wetland structure. However, increasing spatial resolution and sensed spectral bands have created a paradox requiring increasing amounts of expensively collected field data and/or operational time to create homogeneous ROIs and thus meet the requirements of traditional classification approaches. Recently, Costa et al. [4] reiterated the utility of landscape classification using mixed-ROIs as defined in image segmentation. In this study, we contrasted the ability of four different classifiers to characterize a diverse wetland landscape with increasing ROI heterogeneity. The evidence is conclusive that following the Costa et al. [4] approach and using RF provides the highest OA among the four classifiers we explored. We furthermore conclude that the Costa et al. [4] approach with RF provides adequate OA, as defined here by OA of approximately 80% or higher, with ROI sizes of up to 804 m^2^, eight-fold higher than our field-based assessment area. Natural wetland landscapes like the Selenga River Delta tend to be complex, and hence collecting an adequate number of homogeneous ROIs that are representative of the entire wetland landscape can be difficult. The ROI size and heterogeneity increased concurrently in our study area, as we expect it would elsewhere. We therefore do not establish particular thresholds for ROI size as long as there is a fraction of pure ROIs. We do; however, conclude that remote sensing analyses should explore ROIs of several sizes, as thresholds and change points may emerge. ROI size is likely situationally determined as well given that end users could be focused on broad vegetation classes (e.g., open waters, submerged macrophytes, forested wetlands, etc.) or on specific communities (e.g., *Potamogeton*-dominated waters, *Phragmites* patches). These concerns should inform final ROI-size decisions. However, we conclusively state that wetland analyses should embrace ROI heterogeneity rather than attempting to avoid it.

## Figures and Tables

**Table 1.** Description of wetland classes and aquatic habitats from ISODATA unsupervised classifier and subsequently informed by field-based sampling. The habitat data parenthetically noted represents botanical characteristics of the wetland classes with adequate specificity and fidelity to be considered class indicators (see [17] for additional information).

1	Deep water with sand bottom	12	Dense floating vascular (*Nymphoides*)
2	Shallow water with sediment	13	Very dense floating vascular (*Nymphoides*)
3	Shallow water with mud bottom	14	Persistent emergent (*Phragmites*)
4	Very shallow water with sand bottom	15	Persistent emergent (*Bare Soil/Carex*)
5	Shallow water with sand bottom	16	Persistent emergent (*Equisetum*)
6	Submerged aquatic vascular (*Lemna*)	17	Persistent emergent (*Thatch*)
7	Submerged aquatic vascular (*Sparganium*)	18	Persistent emergent (*Carex*)
8	Submerged aquatic vascular (*Ceratophyllum*)	19	Persistent emergent (*Calamagrostis*)
9	Submerged floating vascular (*Nymphoides*)	20	Persistent emergent (*Scolochloa*)
10	Very sparse floating vascular (*Nymphoides*)	21	Persistent terrestrial (*Amoria*)
11	Sparse floating vascular (*Nymphoides*)	22	Shrub/scrub (*Salix*)

**Table 2.** Description of training and validation datasets by region of interest (ROI) size (D is diameter followed by the associated value in m, e.g., D12 is a ROI with a 12-m diameter). There are 228 ROIs.

ROI-ID	Area (m^2^)	% Areal Increase	# of Pure-ROIs	% of Pure-ROIs	# of Mixed-ROIs	% of Mixed-ROIs
D12	113	0	213	93.4	15	6.6
D14	154	36	211	92.5	17	7.5
D15	177	56	126	55.3	102	44.7
D16	201	78	67	29.4	161	70.6
D17	227	101	48	21.1	180	78.9
D18	254	125	39	17.1	189	82.9
D20	314	178	31	13.6	197	86.4
D24	452	300	21	9.2	207	90.8
D28	616	444	13	5.7	215	94.3
D32	804	611	9	3.9	219	96.1
D52	2124	1778	1	0.4	227	99.6

**Table 3.** Classification accuracy (%) by classifier type and region of interest (ROI) size. The highest overall accuracy (OA) for each diameter is in bold and underlined. White text on a dark gray box identifies the highest OA within a given classifier. ROI-IDs that overlap the 95% confidence intervals (CI) for the highest OA for each classifier are highlighted in light grey.

ROI-ID	MLM			GLM			SVM			RF		
	OA	CI		OA	CI		OA	CI		OA	CI	
D12	48.4	47.2	49.6	77.3	76.1	78.4	86.5	85.6	87.4	**87.8**	87.0	88.6
D14	49.6	48.3	50.8	78.7	77.7	79.7	**87.3**	86.3	88.3	87.1	86.2	87.9
D15	55.6	54.2	57.0	77.7	76.6	78.8	86.4	85.4	87.4	**87.2**	86.3	88.2
D16	56.8	55.6	58.0	75.7	74.4	77.0	83.5	82.4	84.7	**85.6**	84.8	86.5
D17	60.5	59.2	61.8	77.0	75.8	78.1	84.4	83.3	85.5	**86.2**	85.4	87.0
D18	60.6	59.3	61.9	75.0	73.8	76.1	82.6	81.6	83.6	**85.9**	85.1	86.8
D20	62.7	61.4	63.9	74.7	73.6	75.8	81.2	80.0	82.4	**84.9**	84.0	85.8
D24	63.7	62.5	65.0	72.3	71.2	73.3	78.0	76.9	79.2	**84.0**	83.1	84.9
D28	62.2	60.9	63.6	70.3	69.2	71.4	77.1	76.0	78.2	**81.7**	80.6	82.8
D32	62.1	60.8	63.3	68.9	67.7	70.1	73.1	71.9	74.2	**79.4**	78.3	80.4
D52	58.5	57.3	59.6	62.2	61.1	63.3	60.2	58.9	61.4	**71.5**	70.4	72.7

Notes: MLM = Multinomial Logistic Model, GLM = Generalized Linear Model, SVM = Support Vector Machine, RF = Random Forest.
